# Effect of Tool Coatings on Machining Properties of Compacted Graphite Iron

**DOI:** 10.3390/mi13101781

**Published:** 2022-10-19

**Authors:** Xiaonan Ai, Jun Tan, Hui Sun, Lu Lu, Zhenming Yang, Zhongguang Yu, Guojun Liao, Shiyong Li, Yilin Jin, Yusheng Niu, Ning He, Xiuqing Hao

**Affiliations:** 1 Weichai Power Co., Ltd., 197 A Fushou East Street, High-Tech Development Zone, Weifang 261061, China; 2 College of Mechanical & Electrical Engineering, Nanjing University of Aeronautics & Astronautics, Nanjing 210016, China; 3 Xiamen Jinlu Special Alloy Co., Ltd., Xiamen 361013, China; 4 Guohong Tools System (Wuxi) Co., Ltd., Wuxi 214101, China

**Keywords:** compacted graphite iron, tool coating, tool wear, machining property, surface roughness

## Abstract

Compacted graphite iron (CGI) has become the most ideal material for automotive engine manufacturing owing to its excellent mechanical properties. However, tools are severely worn during processing, considerably shortening their lifespan. In this study, we prepared a series of cemented carbide-coated tools and evaluated their coating properties in cutting tests. Among all tested coatings, PVD coating made of AlCrN (AC) presented with the best surface integrity and mechanical properties, achieving the best comprehensive performance in the coating test. The AC-coated tool also exhibited the best cutting performance at a low speed of 120 m/min, corresponding to a 60% longer cutting life and the lowest workpiece surface roughness relative to other coated tools. In the cutting test at a high speed of 350 m/min, the CVD double-layer coated tool (MT) with a TiCN inner layer of and an Al_2_O_3_ outer layer had a 70% longer cutting life and the lowest workpiece surface roughness relative to other coated tools.

## 1. Introduction

CGI and gray cast iron (GCI) are two types of cast iron made with different forms of graphite in cast iron [1,2]. Compared with GCI, CGI has better tensile strength, better toughness and lower thermal conductivity. Products made of CGI are lighter-weight, quieter, more wear-resistant and less expensive than GCI products [3,4]. Therefore, they are more suitable for manufacturing large and medium-sized engines. However, the high tensile strength of CGI means that machining requires greater cutting force, high toughness makes it difficult to remove metal and low thermal conductivity leads to difficult heat dissipation and short service life of tools [5,6].

The cutting tool is one of the most important factors with respect to the cutting process of CGI [7]. In the process of machining CGI, tool wear is very serious, and edge breakage sometimes occurs [8]. Tools used to machine CGI should have high hardness, high strength, good wear resistance and sufficient heat resistance and toughness [9]. According to a study that comprehensively considered tool performance and price factors, cemented carbide tools are most well-suited for cutting CGI [10]. With the aim of improving the tool lifespan, researchers optimized the machining CGI machining process, mainly by considering the material [11,12,13,14], tool angle [15,16], tool coating [17,18] and cooling lubrication condition [19,20].

In the current production processes, tool coating is widely used, which can effectively improve the hardness and strength of the tool, in addition to improving the heat resistance and wear resistance of the tool so as to improve its service life. Tooptong S [21] conducted turning tests on uncoated carbide tools and multicoated carbide tools. In their tests, they found that the tool life of coated cutting tool inserts was much longer than that of uncoated cutting tool inserts. Chen M et al. [22] studied the influence of tool coating on cutting temperature using finite element simulation and a dry milling test; the results showed that the cutting temperature of coated tools was 40 ℃ lower than that of uncoated tools. Abdoos M et al. [23] prepared three different low-residual compressive stress Ti_40_Al_60_N-coated tools with coating thicknesses of 5 μm, 11 μm and 17 μm, respectively, and found that the coating with the thickness of 11 μm resulted in the best cutting performance, improving the coating quality and tool life by about 35%. Duchosal A et al. [24] compared single-layer PVD coatings with multilayer CVD coatings and found that CVD coatings were more wear-resistant with a lower friction coefficient than PVD coatings, making them more suitable for CGI processing. The hardness of coating refers to the resistance of the coating surface to external force, which is an important index to measure the mechanical properties of coatings. Oliver W C et al. [25] accurately calculated the mechanical properties of coatings, such as material hardness and elastic modulus, by measuring the relationship curve between indentation load and displacement; they found that the error was within 5%. The abovementioned studies provide a basis for coating testing, which can inform the selection of appropriate coating materials for metal cutting.

Scholars have carried out a number of studies on tool materials, tool coatings and coating characteristics, which are of considerable scientific research value. However, most of these experiments were conducted under laboratory conditions, which differ from real-world conditions, meaning that the obtained results can only be used for reference and lack practical guiding value.

In this study, a series of coated tools was prepared by physical and chemical deposition methods, and tested under realistic conditions. We analyzed the morphology, thickness, mechanical properties and other characteristics of the prepared coatings and conduced cutting experiments according under realistic processing conditions to determine the most suitable coating for CGI cutting applications in order to establish a guiding scheme for actual processing work.

## 2. Experimental Preparation

### 2.1. Coated Tool Preparation

In this study, a YG6 cemented carbide tool (Xiamen Jinlu Special Alloy Co., Ltd., China) was used to processing CGI, which is mainly composed of tungsten carbide (WC) and cobalt (Co) binder. The name YG6 indicates that Co comprises approximately 6% of the total tool volume. The physical properties of the tool are shown in Table 1.

**Figure 1:**
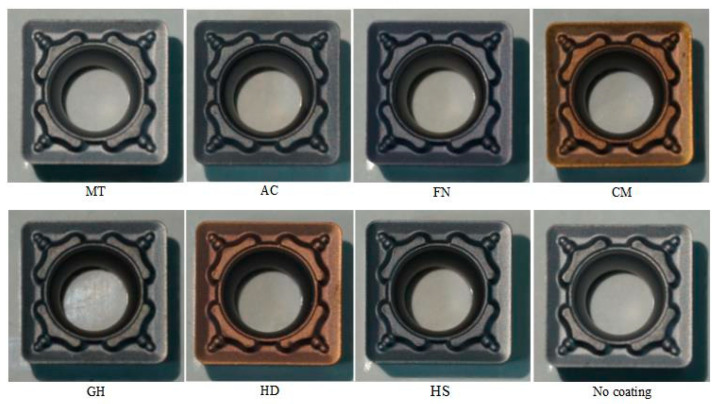
Cutting tool inserts with different coatings.

The coatings were deposited on the cemented carbide substate of an SCMT09T308 tool with a rake angle of 15° and a clearance angle of 7°. Table 2 shows the processing parameters of some of the tested coatings, in all coatings prepared, AC coating was made by Shanghai Tool Works Co., Ltd., Shanghai, China, MT coating was made by Xiamen Jinlu Special Alloy Co., Ltd., Xiamen, China and other coatings prepared were made by Guohong Tools System Co., Ltd., Wuxi, China. Figure 1 shows the cutting tool inserts with different coatings; the MT cutting tool insert was coated with CVD, and other cutting tool inserts were coated with PVD.

### 2.2. Physical Property Test of Tool Coatings

#### 2.2.1. Surface Coating Morphology Inspection

In the test of coating thickness and composition, the coated tool was cut by wire cutting; then, the cut surface was polished. After polishing, the surface was placed into a scanning electron microscope (SEM, ZEISS^TM^-EVO 15, Shanghai, China) and an energy-dispersive X-ray spectroscope (EDS, Horiba^TM^ 7021-H, Shanghai, China) to measure the coating thickness and composition. Because of coatings were thin, in order to reduce the influence of the cemented carbide substrate under the coating on the test results, the indentation depth of the nano indenter must be less than 1/10 of the coating thickness to ensure accurate measurement.

#### 2.2.2. Surface Coating Mechanical Property Test

A common method to measure the hardness of coatings is nanoindentation experiments. In this test, a nanoindentation experiment was conducted using a nano hardness instrument (Hysitron Ti Premier, Bruker Instrument Co., Ltd., Shanghai, China). The loading force was 10 N, and the pressure holding time was 5 s. Measurements were conducted on the same coating deposited on the surface of the cemented carbide block with the same material. The size of the cemented carbide block was 20 mm × 20 mm × 5 mm. The test was repeated 10 times for a single sample, and the average value was reported to ensure the reliability of the data.

**Figure 2:**
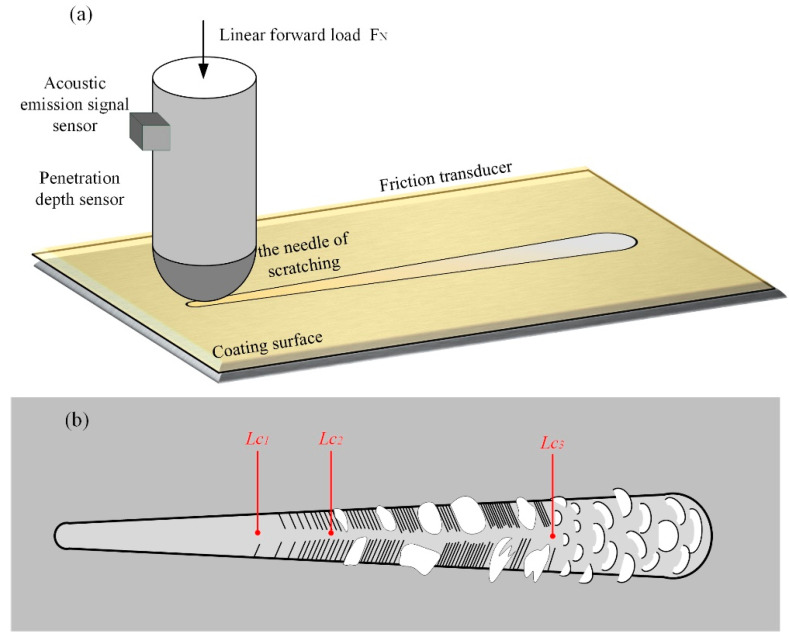
Scratch test: (**a**) schematic diagram; (**b**) schematic diagram of the judgment criteria for binding force.

Adhesion strength an important index of the mechanical properties of coatings, as this factor directly affects the service life and reliability of the coating. The scratch method is the most commonly used technique to characterize the adhesion strength of coatings. A schematic diagram of the scratch test is shown in Figure 2a. A slow, positive load (*F_N_*) was applied to the scratch needle, and the needle tip was uniformly scratched across the coating surface until the coating broke. The critical load (*L_c_*) was determined by detecting the real-time morphology of the scratch and the coating. Figure 2b shows a schematic diagram of the judgment standard of binding force. As the load increased, the coating began to crack; the load at this time was denoted as *Lc*_1_. When the coating began to peel off near the scratch, the load was denoted as *Lc*_2_. When the load increased further and the coating spared off in a large area, the load denoted as *Lc*_3_. In this study, *Lc*_2_ was selected to characterize the binding force of the coating, and *Lc*_3_ was selected to characterize the membrane breaking force of the coating. In this test, a scratch tester (RST^3^, Anton-Paar (Shanghai, China) Trading Co., Ltd., Shanghai, China) was used, with a load range of 1–120 N, a load increase speed of 238 N/min, a scratch distance of 5 mm and a moving speed of 10 mm/min.

### 2.3. Workpiece Material

**Figure 3:**
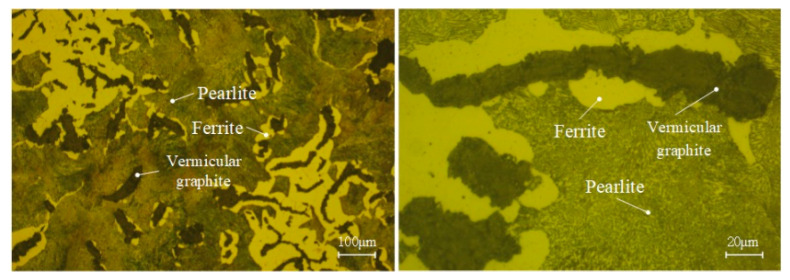
Crystal phase diagram of CGI.

The Rut450 CGI model was used in this turning test. A crystal phase diagram of the material is shown in Figure 3, in which vermicular graphite, ferrite and pearlite can be clearly observed. Other physical properties of the material are shown in Table 3.

### 2.4. Cutting Experiments

A CNC lathe (BAOJI SK50P) was used in a dry cutting experiment to evaluate the cutting performance of the coated tools. According to the processing procedures, coated and uncoated tools were used to cut CGI at 120 m/min (low speed) and 350 m/min (high speed) with a feed rate of 0.14 mm/r and a cutting depth of 0.1 mm. 

The cutting performance of the coated tools was judged according to three factors: the machined surface roughness, the effective cutting life of the tool and tool flank wear. Surface roughness (Ra) was measured using handheld surface roughness meter (Mahr perthometer M1, Mahr Precision Metrology (Suzhou) Co., Ltd., Suzhou, China); the average roughness value of eight measured machined surfaces was taken as the surface roughness value of the machined workpiece.

## 3. Results and Discussion

### 3.1. Coating Morphology

**Figure 4:**
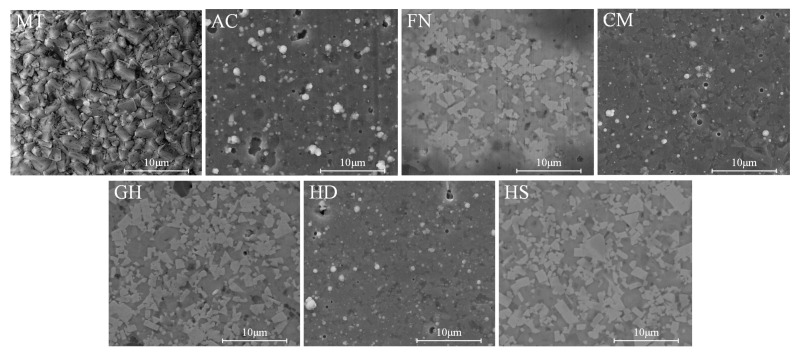
Images of the morphology of the coating surfaces.

**Figure 5:**
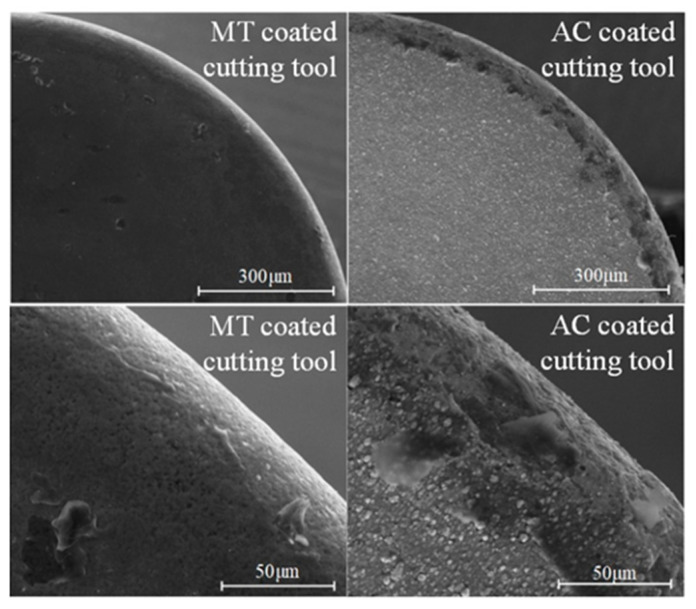
Rake face images of MT- and AC-coated cutting tools.

Coating morphology is an important indicator of the superiority of reactive coatings, which can affect the adhesion between surface coatings and substrates. For some PVD coatings, a small number of particles and holes appeared on the surface of the prepared coating, which may have been caused by molten metal droplets when the PVD coating was deposited from the cathode target to the cemented carbide substrate surface. Surface morphology images of the tested coatings are shown in Figure 4. There were no obvious defects, and each coating was dense. Taking MT and AC coatings as examples, as shown in Figure 5, the working part of the tool had no cracked edges after coating. The membrane layer on the coating surface was complete, without obvious defects, and no membrane layer had fallen off, satisfying processing requirements.

### 3.2. Thickness and Coating Composition

**Figure 6:**
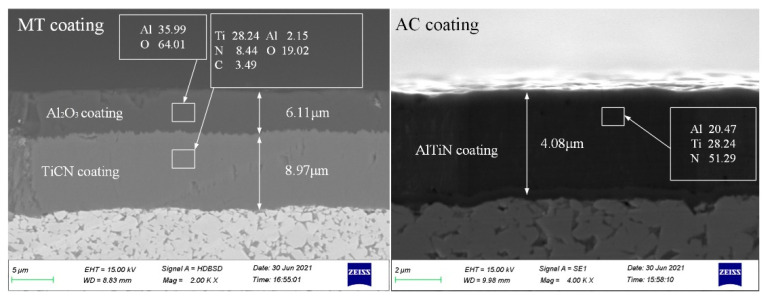
Thickness and composition of MT and AC coatings.

The thickness and composition of the coatings considerably influence the equivalent stress of the tool, thus affecting tool wear during cutting. Images of the thickness and composition of thicker MT coating and thinner AC coating are shown in Figure 6. Table 4 shows the percentage thickness values and compositions of different types of coatings. The MT coating is double-layer coating, resulting in a higher overall thickness. The bottom layer is a TiCN coating (about 9 μm), and the outer layer is an Al_2_O_3_ coating (about 6 μm); this structure improved the hardness of the tool, providing improved lubricity. AC, GH and HS coatings have a similar composition, all containing AlCrN. The FN coating was contained AlTiN. The CM coating contained AlSiN. The HD coating contained AlTiN-TiCrSiN. The Al_2_O_3_ layer formed in these coatings can effectively improve the high-temperature life of the tool, making it suitable for dry or semidry cutting. The hardness and oxidation resistance of the coating can be adjusted by changing the aluminum content and coating structure. For example, the oxidation resistance of the coating can be improved by increasing the aluminum content and adopting a nanostructure or microalloying.

### 3.3. Mechanical Properties of Coatings

According to the obtained loading and unloading curve, the hardness of the material could be obtained using the following formula:

(1) $$H=\frac{P_{max}}{A}$$

where *H* is the hardness of the material, *P_max_* is the maximum load during loading and *A* is the projected area of the indentation.

The elastic modulus is generally used to express the toughness of the coating. The elastic modulus of the coating can be calculated using the following formula:

(2) $$E_{r}=\frac{1-\gamma^{2}}{E}+\frac{1-\gamma_{i}^{2}}{E_{i}}$$

(3) $$S=\frac{dP}{dh}=\frac{2}{\sqrt{\pi }}E_{r}\sqrt{A}$$

where $E_{r}$ is the equivalent elastic modulus, *E* is the elastic modulus of the tested coating, *γ* is the Poisson’s ratio of the tested coating, $E_{i}$ is the elastic modulus of the indenter material, $\gamma_{i}$ is the Poisson’s ratio of the indenter material and *S* is the slope of the upper end of the unloading curve.

**Figure 7:**
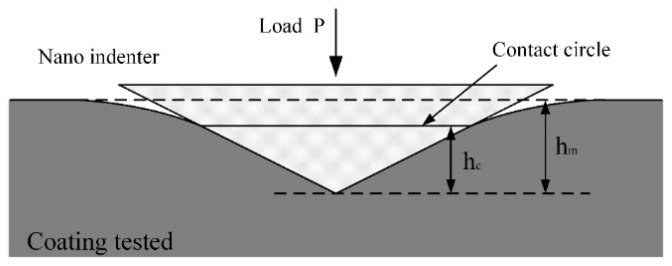
Profile during nanoindentation test.

**Figure 8:**
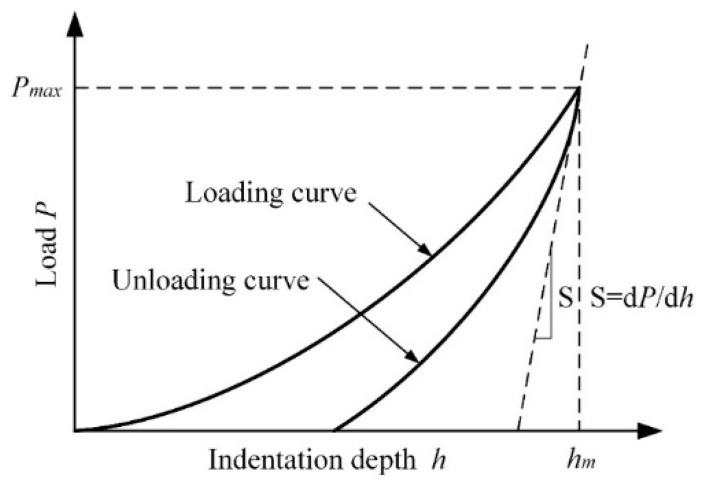
Loading–unloading curve of the nanoindentation test.

The indentation projection area (*A*) is generally calculated using the following empirical formula:

(4) $$A=C_{0}h^{2}+C_{1}h+C_{2}h^{\frac{1}{2}}+C_{3}h^{\frac{1}{4}}+\ldots$$

where *h* is the indentation depth of the indenter, and $C_{0}$ and $C_{1}$… are constants. Because the coating has a sinking effect after loading (Figure 7), according to the research of Oliver W C et al. [25], the real contact depth of the indenter is expressed as follows:

(5) $$h_{c}=h_{m}-\varepsilon \frac{P_{max}}{S}$$

where $h_{c}$ is the true contact depth of the indenter. $h_{m}$ is the maximum indentation depth, and ε is the geometric constant of the conical indenter (in our experiment, *ε* = 0.72). *P_max_*, $h_{m}$ and *S* can be obtained from the loading-unloading curve (Figure 8); then, the hardness (*H*) and elastic modulus (*E*) of the coating can be calculated.

**Figure 9:**
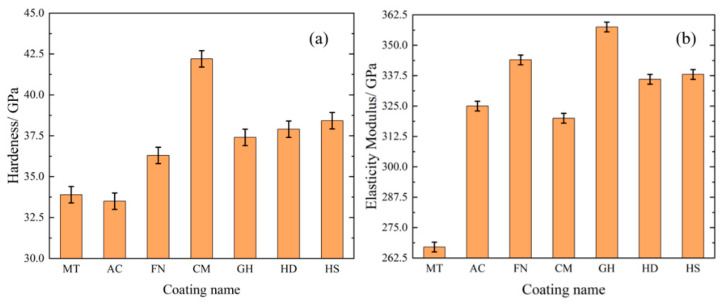
Hardness and elastic modulus of different coatings.

Ensuring a high surface hardness of the coating is one of the best ways to improve the tool life. Generally speaking, under constant conditions, the higher the hardness of the material or surface, the longer the service life of the tool [26,27]. The hardness (*H*) of different kinds of coatings are shown in Figure 9a, and elastic modulus (*E*) of different kinds of coatings are shown in Figure 9b. The CM coating had the highest hardness, corresponding to best ability to resist external damage. The hardness of the MT coating was very low, and the hardness of AC coating was the lowest, corresponding to an increased probability of edge collapse during cutting. In terms of the elastic modulus of the coating, the MT coating had the lowest elastic modulus among all the tested coatings, which was far lower than that of all other coatings. The CM coating had the lowest elastic modulus, except MT. The FN and GH-008 coatings had the highest elastic modulus and the best coating toughness, indicating that they were not prone to brittle fracture.

**Figure 10:**
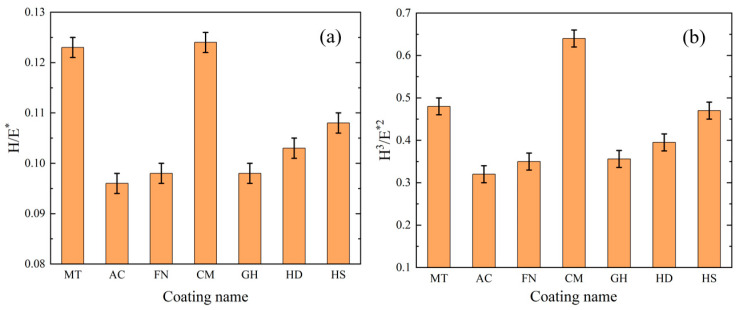
H/E* and H^3^/E*^2^ values of different coatings.

In addition to the hardness and elastic modulus of the coating, the H/E* value and H^3^/E*^2^ value of the coating are positively correlated with the plastic deformation and fracture tension [28,29]; $E^{*}=E/\left(1-\gamma^{2}\right)$, and *γ* is Poisson’s ratio. The H/E* value of the coatings after calculation are shown in Figure 10a, and H^3^/E*^2^ value of the coatings after calculation are shown in Figure 10b. The H/E* value and H^3^/E*^2^ value of CM were 0.1238 and 0.6439, indicating that this coating exhibited the best bearing performance and fracture toughness, respectively. The ratios of the MT coating were 0.1213 and 0.5014, which were second only to CM coating. The H/E* and H^3^/E*^2^ ratios of the AC coating were 0.0967 and 0.3131, respectively, which were the lowest among all tested coatings.

**Figure 11:**
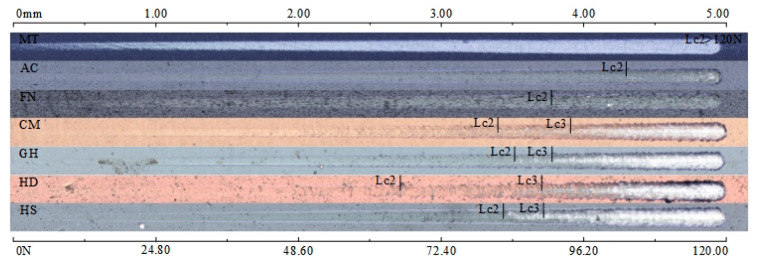
Scratch morphology of each coating surface.

**Figure 12:**
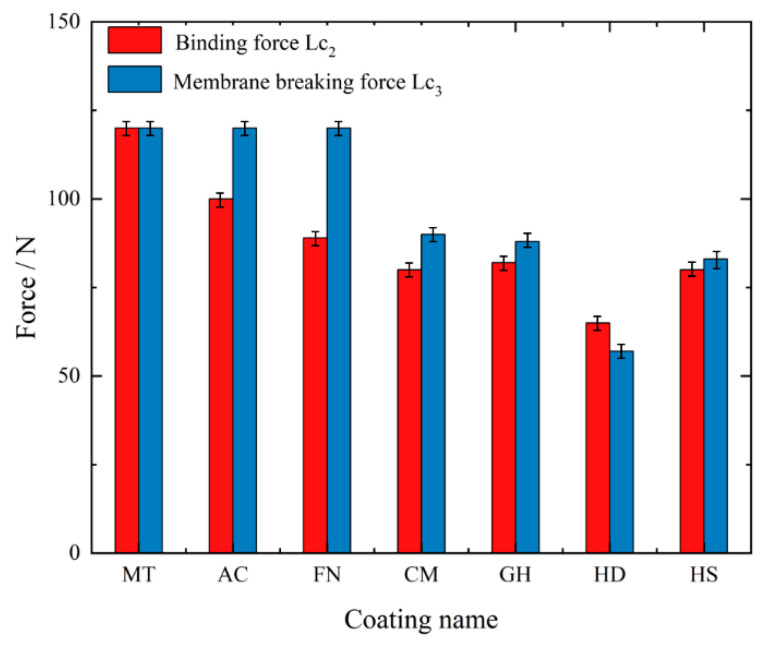
Binding force and membrane breaking force of each coating.

The scratch test results of the coatings are shown in Figure 11. The binding force (*Lc*_2_) and membrane breaking force (*Lc*_3_) of each coating are shown in Figure 12. For the MT coating, due to its thick CVD coating and high hardness of the Al_2_O_3_ coating on the surface, the binding force and membrane breaking force of the coating were both higher than 120 N. The AC coating was the thickest PVD coating, with a membrane breaking force of more than 120 N and a binding force of 100.92 N—the highest among all tested coatings. For FN coating, the membrane breaking force was more than 120 N, but its binding force was 88.38 N, slightly lower than that of the AC coating. For the CM, GH and HS coatings, the binding force and membrane breaking force values were roughly equal, at approximately 80 N and 87–91 N, respectively. The binding force and membrane breaking force of the HD coating were 62.97 N and 86.71 N, respectively—the lowest of all tested coatings.

### 3.4. Cutting Performance Test of Coated Tools

The cutting experiment was carried out under actual factory processing conditions. During actual production and processing, in order to achieve the highest economic benefits under the premise of ensuring the product quality, the product quality judgment standard is the processed surface roughness. The cutting tool with the highest cutting life at the same price is therefore considered to have the highest economic benefit when the surface roughness of the machined workpiece reaches the standard. According to the processing procedures, tests were conducted at a low speed (120 m/min) and at a high speed (350 m/min). Machined surface roughness and flank wear are important parameters to evaluate tool wear. In this test, when the processed surface roughness (Ra) is higher than 1.6 μm under the high-speed condition and higher than 2.0 μm under the low-speed condition, the tool is considered to satisfy the wear and scrap standard, and the cutting length is recorded at this time.

#### 3.4.1. Cutting Experiment under Low-Speed Condition

**Figure 13:**
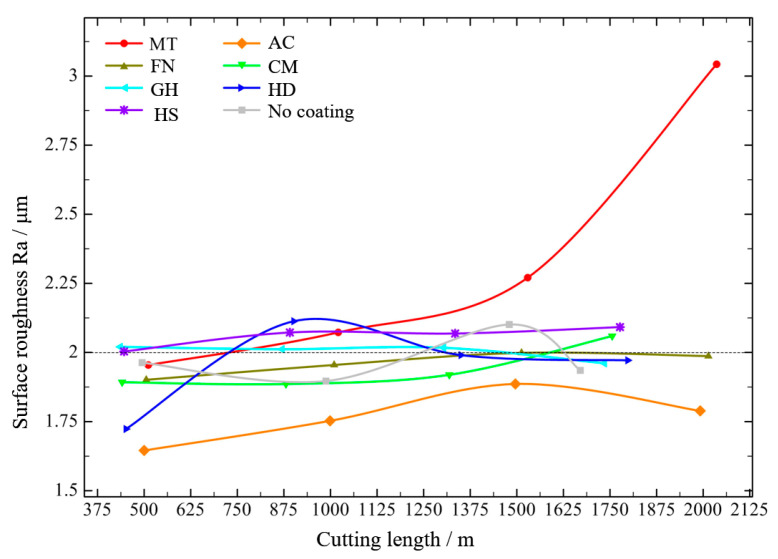
Surface roughness of the workpiece of different coatings at low speed.

The surface roughness of the machined surface at low speed is shown in Figure 13. Among all tested coatings, the AC coating was determined the best coating for cutting CGI at low speed. The surface roughness value for about 500 m was close to 1.6 μm at the beginning, but the maximum roughness value did not exceed 2 μm with increased cutting length. The MT coating achieved the worst cutting performance, and when the cutting distance exceeded 2000 m, the surface roughness exceeded 3 μm. Other coatings achieved similar cutting performance, that were; with increased cutting length, the surface roughness of the workpiece was about 2 μm.

**Figure 14:**
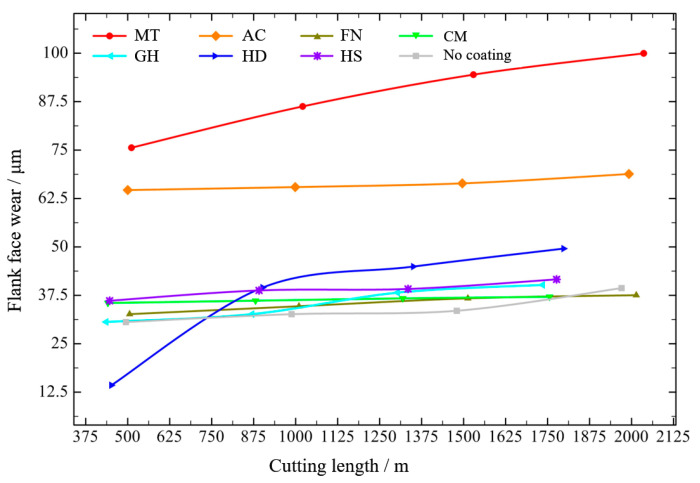
Tool flank face wear of different coatings at low speed.

The flank face wear values of different surface-coated tools are shown in Figure 14. Under the low-speed condition, the wear of each tool was very slight and still in the normal wear state, and wear increased with increased in cutting length. The MT coating exhibited the most wear, reaching 99.97 μm after cutting 2000 m, whereas the AC coating achieved the best performance and higher wear, reaching 68.82 m after cutting about 2000 m. Other coatings exhibited similar wear, ranging from 37 μm to 50 μm.

**Figure 15:**
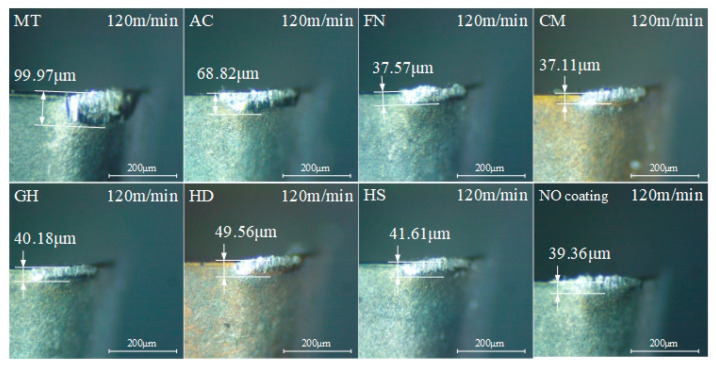
Tool flank wear of different coatings after cutting 1700 m at low speed.

According to the above results, when different coated tools were used to cut compacted graphite iron at low speed, when the cutting length reached about 1700 m, there was an obvious gap in both the workpiece surface roughness and the tool flank wear, which may indicate that a 1700 m cutting length of cemented carbide coated tools is a turning point when gaps between different coating types are evident when machining compacted graphite iron. The flank wear results of the coated tools after cutting about 1700 m are shown in Figure 15. The tool wear was very slight, with no obvious damage to the tool coatings; the cemented carbide substrate was not exposed, and no yellow ablative trace was found, indicating the normal wear stage.

In summary, the AC coating exhibited the best machined surface roughness and the longer processing life among all tested coatings during low-speed processing of CGI. Huang J [30] studied the effect of cutting speed on tool cutting force and found that during low-speed cutting (under 250 m/min), the cutting force decreases with increased cutting speed. Chen X [31] studied the impact tool toughness on the cutting process and found that tools with higher elastic modulus exhibited better toughness under the condition of low hardness, indicating better wear resistance. According to the information presented in Figure 9, the AC coating exhibited a greater elastic modulus under lower hardness conditions, indicating that the AC coating has better toughness. Thus, it has better wear resistance and better cutting performance under the action of greater cutting force during low-speed cutting. The cutting performances of other coatings are similar, whereas the MT coating is not suitable for low-speed processing of CGI.

#### 3.4.2. Cutting Experiment under High-Speed Condition

**Figure 16:**
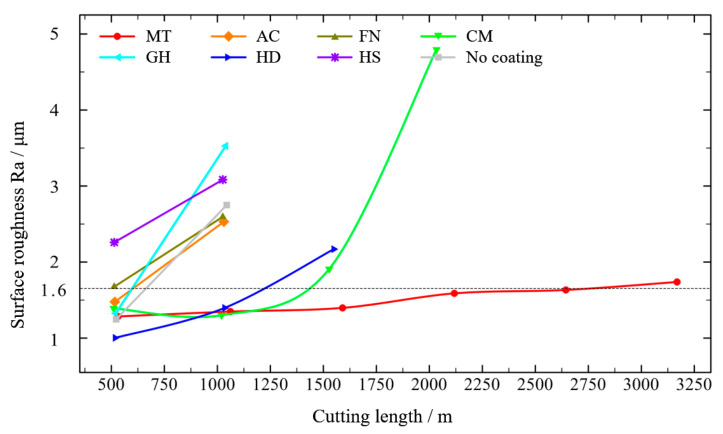
Surface roughness of the workpiece with different coatings at high speed.

The surface roughness of the machined surface at high speed (350 m/min) is shown in Figure 16. With increased cutting length, tool wear affect the surface quality after machining. The surface roughness (Ra) of the MT-coated tool increased slowly, and the surface roughness exceeded 1.6 μm when the cutting distance exceeded 2250 m. The performance of the CM and HD coatings were similar; the surface roughness still met the requirements when the cutting distance was close to 1000 m, and the surface roughness exceeded 1.6 μm when the cutting distance exceeded 1500 m. The performances of the AC and GH coatings were similar to that of the tool without coating, meeting the requirements when the cutting distance was about 500 m. Coating can alleviate wear of the tool, but it cannot improve the surface roughness. The effective cutting distances of the FN and HS coatings were less than 500 m.

**Figure 17:**
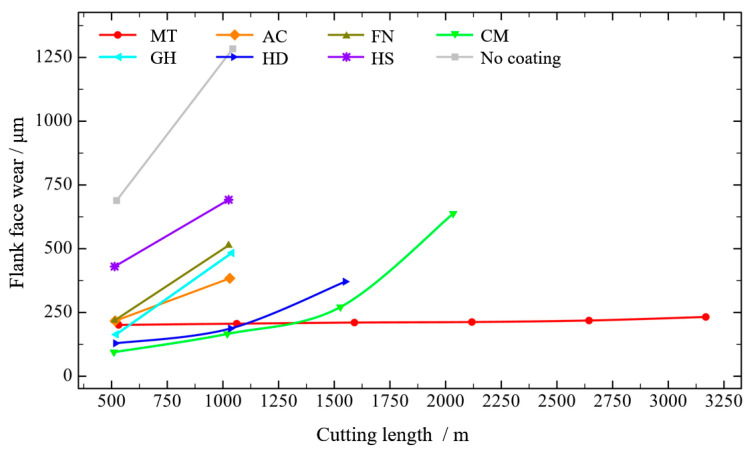
Tool flank wear of different coatings at high speed.

The flank wear values after cutting with different coatings are shown in Figure 17. When the cutting length of the uncoated tool was close to 500 m, flank wear reached 689.03 μm. When the cutting length exceeded 1000 m, the flank wear reached 1283.90 μm, indicating a serious degree of wear. With increased cutting length, the flank wear of the MT-coated tool increased slowly. When the surface roughness was close to 1.6 μm, the flank wear value was about 218.57 μm, indicating that the tool had good wear resistance. The wear of the flank face with CM and HD coatings was 165.95 μm and 187.28 μm, respectively, when the cutting distance was just over 1000 m. Tool wear increased faster when the cutting distance was close to 1500 m to 271.25 μm and 370.85 μm with CM and HD coatings, respectively, and the cutting surface roughness also exceeded 1.6 μm. The flank wear values of AC, FN and GH coatings were low when the cutting distance was 500 m, increasing rapidly when the cutting length was more than 1000 m, resulting in excessive surface roughness. When the cutting length of the HS coating tool was close to 500 m, the flank wear reached 430.27 μm, indicating the worst coating resistance.

**Figure 18:**
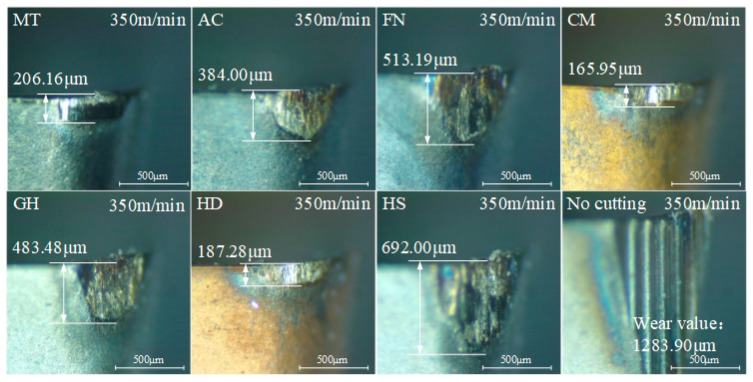
Tool flank wear of different coatings after cutting 1000 m at high speed.

The flank wear of the coated tools after cutting for 1000 m at 350 m/min is shown in Figure 18. Although the surface roughness of the uncoated cemented carbide tool met the requirements for the first 500 m, as shown in the Figure 16, the flank face wear was very serious, and plowing occurred due to extrusion. For the cutting tool inserts with a low wear degree with MT, CM and HD coatings, the coatings were damaged, but the area and depth of the damaged part were minimal and still within the normal wear stage of the tool. The MT-coated tool had the least flank wear, which was 522% less than that of the uncoated tool, with a cutting life of 2000 m more than that of the uncoated tool. For other types of coated tools, the damage area of the coating on the flank face was larger, and there were still yellow ablation traces on the surface of the contact region. Moreover, a large number of sparks were observed during the cutting process, proving that the high-temperature resistant coating on the surface had been damaged, possibly because during high-speed cutting, the high temperature generated by the friction between the cemented carbide and the workpiece ignited in the process of metal processing, resulting in serious tool wear and affecting the quality of the machined surface.

In summary, under the high-speed condition, the growth trend of tool flank wear and surface roughness is basically the same, and the tool surface coating can effectively reduce tool wear and improve the surface quality after machining. The MT coating achieved the best workpiece surface roughness, the lowest tool wear and the longest tool life, making it the most suitable coating for processing of CGI among tested coatings. And The HS coating is not suitable for processing of CGI. Therefore, we speculate that during the high-speed machining process, owing to the small feed and short cutting depth, the cutting force is considerably reduced, with a more intense interaction between the tool and the workpiece, making the tool more prone to damage. The H/E* and H^3^/E*^2^ values of the MT coating were higher, indicating that the MT coating is more resistant to deformation and fracture. According the information presented in Figure 12, the MT coating has the highest binding and membrane breaking force, corresponding to the best bonding performance with the cemented carbide matrix and the ability to resist impact damage under high-speed machining.

## 4. Conclusions

In this study, seven PVD- and CVD-coated tools were prepared, and the properties of their coating were tested under actual CGI processing conditions. Then, the corresponding cemented carbide-coated tools were evaluated in cutting experiments under low-speed and high-speed conditions. The main conclusions of this study are as follows:

(1) The AC coating was the best tool coating for machining of CGI at a low speed of 120 m/min. In the low-speed cutting experiment, with increased cutting length, the surface roughness value of most coatings exceeded 2 μm when the cutting length reached 1250 m. However, when cutting length exceeded 2000 m, the surface roughness value of the AC-coated tool did not exceed 2 μm, with a 60% longer cutting life than other coated tools, achieving the best cutting performance among all tested coatings. The AC coating was an AlCrN series PVD coating, with low coating hardness, low elastic modulus and the lowest H/E* and H^3^/E*^2^ values, indicating the low coating hardness and toughness. Compared with the CVD coating, its denser PVD surface coating can resulted in a better resistance at low speed. Compared with other PVD coatings, the lower hardness and toughness of the coating can make it wear less during low-speed machining.
(2) The MT coating was the best tool coating for machining of CGI at a high speed of 350 m/min. In the high-speed cutting experiment, the flank wear of the MT-coated tool increased slowly with increased cutting length. When the machined surface roughness was close to 1.6 μm, the flank wear value was about 218.57 μm, and when the cutting distance exceeded 2250 m, the surface roughness exceeded 1.6 μm. Its cutting life exceeded that of other coatings by at least 70%. The MT coating was a CVD coating; the inner layer was TiCN with a thickness of about 9 μm, and the outer layer was Al_2_O_3_ with a thickness of about 6 μm. It had relatively low hardness and the lowest elastic modulus of all tested coatings. However, the H/E* and H^3^/E*^2^ values of the MT coating were high, which proved that the coating had high toughness and wear resistance. As a CVD coating, the MT coating had a high bonding strength and high toughness and wear resistance, indicating that it can better protect the tool than other coatings under the intense action of high speed.

## Figures and Tables

**Table 1:** Physical properties of YG6 cutting tools.

Physical Property	Density (g/cm^3^)	Young’s Modulus (GPa)	Poisson’s Ratio	Thermal Conductivity (W/mK)	Specific Heat (J/g·K)	Thermal Comparator
Value	14.85	640	0.25	79.6	0.176	0.9

**Table 2:** Processing parameters of tested coatings.

Name	Work Pressure (Pa)	Deposition Temperature (°C)	Target Current (A)	Deposition Time (min)	Bias Voltage (V)
AC	3.5	480	140	64	40/100
FN	3.2	450	200	78	40
CM	3.5	480	125	35	40
GH	3	480	110	40	100
HD	4	480	120	65	40
HS	3.5	480	110	35	100

**Table 3:** Physical properties of CGI.

Physical Property	Density (g/cm^3^)	Young’s Modulus (GPa)	Poisson’s Ratio	Thermal Conductivity (W/mK)	Thermal Diffusivity (mm^2^/s)	Specific Heat (J/g·K)
Number	7.1	133	0.26	36.28	0.996	0.471

**Table 4:** Compositions of different types of coatings.

Coating Name	Coating Thickness (μm)		Elemental Content (%)						
			Al	Cr	Ti	Si	N	O	C
MT (double-layer)	6.11	(outer)	35.99%	-	-	-	-	64.01%	-
	8.97	(inner)	2.15%	-	45.41%	-	8.44%	19.02%	34.99%
AC	4.08		36.30%	17.60%	-	-	46.10%	-	-
FN	2.14		20.47%	-	28.24%	-	51.29%	-	-
CM	1.86		-	-	34.23%	14.41%	51.36%	-	-
GH	2.21		35.21%	14.86%	-	-	49.93%	-	-
HD	2.26		17.63%	8.85%	20.68%	3.47%	49.37%	-	-
HS	1.48		33.94%	15.28%	-	-	50.78%	-	-
